# Prevention of exercised induced cardiomyopathy following Pip-PMO treatment in dystrophic *mdx* mice

**DOI:** 10.1038/srep08986

**Published:** 2015-03-11

**Authors:** Corinne A. Betts, Amer F. Saleh, Carolyn A. Carr, Suzan M. Hammond, Anna M. L. Coenen-Stass, Caroline Godfrey, Graham McClorey, Miguel A. Varela, Thomas C. Roberts, Kieran Clarke, Michael J. Gait, Matthew J. A. Wood

**Affiliations:** 1 Department of Physiology, Anatomy and Genetics, University of Oxford, South Parks Road, Oxford, UK, OX1 3QX; 2 Medical Research Council, Laboratory of Molecular Biology, Francis Crick Avenue, Cambridge, CB2 0QH, UK; 3 Department of Molecular and Experimental Medicine, The Scripps Research Institute, 10550 NTorrey Pines Road, La Jolla, CA 92037, USA; 4 AstraZeneca R&D, Discovery Safety, Drug safety and Metabolism, Alderley Park, Macclesfield, SK10 4TG, UK

## Abstract

Duchenne muscular dystrophy (DMD) is a fatal neuromuscular disorder caused by mutations in the *Dmd* gene. In addition to skeletal muscle wasting, DMD patients develop cardiomyopathy, which significantly contributes to mortality. Antisense oligonucleotides (AOs) are a promising DMD therapy, restoring functional dystrophin protein by exon skipping. However, a major limitation with current AOs is the absence of dystrophin correction in heart. Pip peptide-AOs demonstrate high activity in cardiac muscle. To determine their therapeutic value, dystrophic *mdx* mice were subject to forced exercise to model the DMD cardiac phenotype. Repeated peptide-AO treatments resulted in high levels of cardiac dystrophin protein, which prevented the exercised induced progression of cardiomyopathy, normalising heart size as well as stabilising other cardiac parameters. Treated mice also exhibited significantly reduced cardiac fibrosis and improved sarcolemmal integrity. This work demonstrates that high levels of cardiac dystrophin restored by Pip peptide-AOs prevents further deterioration of cardiomyopathy and pathology following exercise in dystrophic DMD mice.

Duchenne muscular dystrophy (DMD) is a severe, debilitating, muscle wasting disorder caused by mutations that disrupt the reading frame of dystrophin mRNA, thus preventing production of this essential structural protein^1^. Cardiomyopathy is prominent in DMD patients and manifests with left ventricular dilation and hypertrophy, decreased fractional shortening and electrocardiogram (ECG) abnormalities^2^,^3^,^4^,^5^. As the disease progresses, dilated cardiomyopathy (DCM) develops, a condition prevalent in all DMD patients by adulthood^3^. While respiratory complications are still the major cause of death amongst DMD patients^6^, cardiomyopathy contributes significantly to premature death^7^. A number of cardiac pharmacotherapies are in clinical use^8^, however, no therapy is currently capable of correcting the underlying genetic defect and restoring dystrophin protein in the heart.

A multitude of experimental therapies have been investigated in an effort to restore^9^,^10^,^11^ or substitute^12^,^13^ for dystrophin. Antisense oligonucleotides (AOs) act to modulate splicing of dystrophin pre-mRNA to generate a truncated, but functional dystrophin protein^14^,^15^,^16^. 2′ O-Methyl phosphorothioate (2OMePS) and phosphorodiamidate morpholino oligomer (PMO) AO chemistries have shown great promise in recent clinical trials^17^,^18^,^19^,^20^, with the most recent study reporting encouraging improvements in the 6 minute walk test following continuous treatment with Drisapersen (2OMePS) for 48 weeks^21^. Despite this, both chemistries have demonstrated both limited and variable ability to restore dystrophin, with negligible activity in heart^22^,^23^.

A powerful approach to improve AO potency is the conjugation of cell penetrating peptides to uncharged AOs, such as PMO^24^,^25^,^26^,^27^. While early generation peptide-PMOs were weakly active in heart, they were shown to have an influence on heart function in *mdx* mice displaying a mild cardiac phenotype^27^,^28^. Recently we have developed a novel series of highly active peptides known as Pip's (PMO internalising peptides) for PMO delivery^29^. This series of peptides comprise a hydrophobic central core with 2 flanking arginine-rich domains. This combines the improved skeletal muscle delivery capacity observed with the arginine rich B-peptide, with a hydrophobic sequence that dramatically enhances efficacy to create a new innovative series of peptides^30^. Previous studies showed that the hydrophobic region is critical for the delivery to the heart tissue as other arginine-rich peptides with a shorter hydrophobic region failed to achieve this. Progressive evolution of this peptide series through structure-activity studies has identified novel peptides with dramatically enhanced cardiac dystrophin restoration^30^,^31^,^32^. Pip6-PMO is currently the most potent class of peptide-PMO, demonstrating high levels of dystrophin protein restoration following a single low intravenous dose, most notably in cardiac muscle.

Here we sought to determine the benefit on cardiac function and pathology offered by Pip6-PMO conjugates which restore high levels of dystrophin in heart. To do this we modelled the treatment of DMD cardiomyopathy by repeat treating dystrophic *mdx* mice in which a moderate cardiomyopathy was induced by forced exercise over a twelve week period. Using stringent statistical measures, our results demonstrate that restoring high levels of dystrophin in heart with Pip6-PMO was sufficient to prevent exercise-induced progression of cardiomyopathy and to improve multiple indices of cardiac pathology.

## Results

### Repeat doses of Pip6f-PMO restore high levels of dystrophin protein in *mdx* mouse hearts

**Figure 1:**
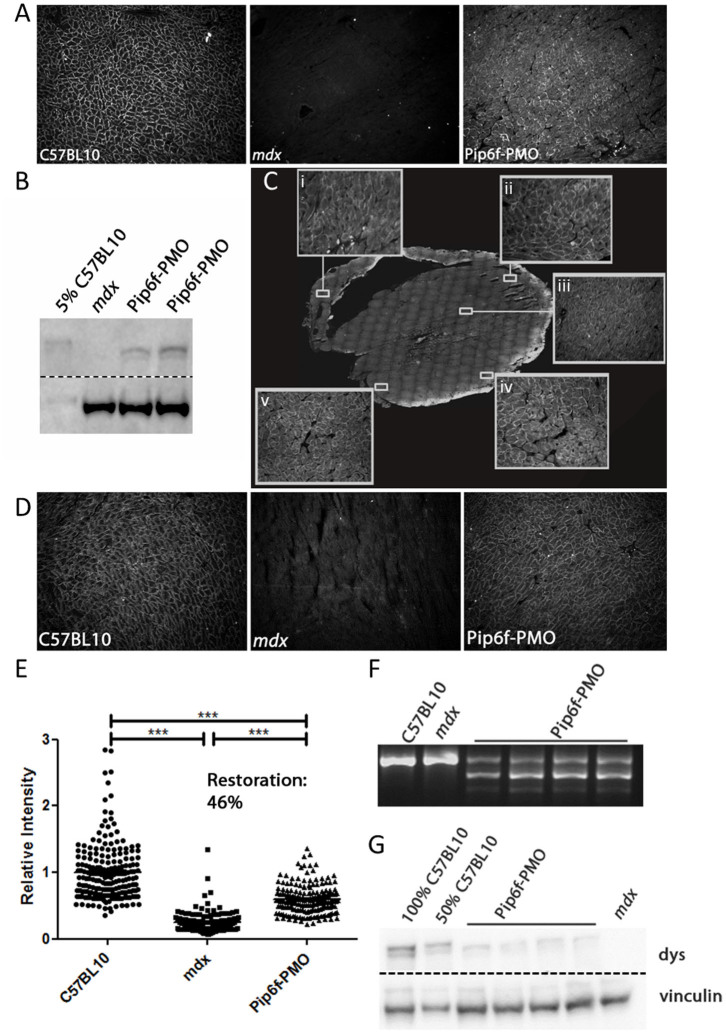
Dystrophin restoration in heart following Pip6f-PMO treatment in *mdx* mice. (*A*) Representative image of dystrophin immunohistochemical staining in heart following single 10 mg/kg Pip6f-PMO IV administration in *mdx* mice. (*B*) Dystrophin western blot of treated *mdx* hearts relative to 5% C57BL/10 following a single 10 mg/kg Pip6f-PMO administration. Approximately 5% dystrophin restoration observed. All samples run under the same experimental conditions and on the same SDS gel. Dotted line indicates where image was cropped. (*C*) Mice then underwent 4 daily injections followed by additional administrations every two weeks. Dystrophin immunohistochemical staining of treated heart with inserts indicating higher magnification of designated areas namely the right ventricle (RV) wall (*C, i*), outer left ventricle (LV) wall at apex (*C, ii*), inner myocardium (*C, iii*), and base (*C iv* and *v*) of heart. (*D*) Representative images of immunohistochemical staining of dystrophin protein in exercised C57BL/10, *mdx* and Pip6f-treated *mdx* mouse hearts. (*E*) Quantification of immunohistochemical staining in hearts (from *D*) following multiple administrations. Dystrophin expression is determined relative to laminin co-stain. The scatter plots show the normalised relative intensity values for each region of interest. Statistical significance was determined using ANOVA followed by Games-Howell post-hoc test to correct for variance heterogeneity (*** = *P* < 0.001, ** = *P* < 0.01, * = *P* < 0.05). (*F*) Representative images of reverse-transcriptase (RT) PCR illustrating Δ23 splicing in heart Pip6f-PMO treated cohort. All samples run under the same experimental conditions and on the same agarose gel. RT-qPCR was also performed (see Supplementary Fig. 3 B) which resulted in 32.3% *Dmd* transcripts lacking exon 23, following normalisation to exon 20–21 (SEM 3.1). (*G*) Representative images of western blots for Pip6f-PMO treated heart. For western blots, 10 μg of protein was loaded and dystrophin (dys) was quantified relative to vinculin loading control. All samples run under the same experimental conditions and on the same SDS gel. Dotted line indicates where image was cropped. For full agarose and SDS gels see Supplementary Fig. 1, 5&6.

The widely used dystrophic *mdx* mouse model has a natural mutation in exon 23 of the *Dmd* gene which generates a premature termination codon. AOs directed towards exon 23 induce exon skipping and allow restoration of a shorter, but functional dystrophin protein. The most recent screen of Pip6-PMO conjugates revealed 2 lead candidates, Pip6a- and Pip6f-PMO, which were capable of restoring high dystrophin protein levels following a single administration. Pip6f-PMO was chosen for the purpose of this study and a single lower dose of 10 mg/kg, delivered intravenously (IV), was performed to confirm effective dystrophin restoration in heart. Figure 1 displays dystrophin protein restoration in *mdx* heart muscle fibres as measured by immunohistochemical staining (Fig. 1A) and western blotting (Fig. 1B and Supplementary Fig. 1). The single, low dose treatment resulted in 5% dystrophin protein restoration in heart relative to C57BL/10 control. Therefore a longer treatment regimen was initiated whereby 12 week old *mdx* mice received IV administrations of 10 mg/kg on 4 consecutive days followed by 5 doses every 2 weeks (Supplementary Fig. 2). Immunohistochemical staining of the treated *mdx* hearts revealed widespread and homogenously distributed dystrophin protein restoration (Fig. 1C). This is indicated by the higher magnification inserts showing right ventricle (RV) wall (Fig. 1 C, i), outer left ventricle (LV) wall from apex (Fig. 1 C, ii) to base (Fig. 1 C iv and v) and inner myocardial dystrophin protein restoration (Fig. 1 C, iii). Representative images of dystrophin restoration in treated hearts relative to the C57BL/10 and *mdx* controls are also shown (Fig. 1D). Quantification of dystrophin staining was determined relative to a laminin co-stain. The dystrophin intensity values of multiple regions of interest, relative to the corresponding intensity value of laminin, were normalised to C57BL/10. This generated a recovery score of 46% in the hearts of the treated cohort (Fig. 1E). In addition, RT-qPCR revealed high levels of dystrophin transcript (32% of *Dmd* transcripts lacking exon 23, see Supplementary Fig. 3 B; RT-PCR also showed high Δ23 exon skipping, see Fig. 1F), and western blotting quantification indicated 28% protein restoration in heart tissue (Fig. 1G and Supplementary Fig. 3 C).

Dystrophin was also restored in the diaphragm and other accessory respiratory muscles, intercostal (IM) and sternomastoid (SM) as well as *tibialis anterior* (TA) of the lower limb. Again immunohistological staining demonstrated extensive dystrophin protein restoration (Supplementary Fig. 3 A) with exceptionally high recovery scores between 74–104% (Supplementary Fig. 4). The RT-qPCR (Supplementary Fig. 3 B) and western blot quantification (Supplementary Fig. 3 C&6) exhibited similar results with very high dystrophin transcript levels (77–86%) and between 58–101% restoration of dystrophin protein in the diaphragm, TA, IM and SM muscles. RT-PCR also showed complete skipping of exon 23 in these muscles (Supplementary Fig. 5). This demonstrates that the repeated low-dose Pip6f-PMO administration protocol successfully restored high levels of dystrophin in skeletal and cardiac muscles.

### Exercised *mdx* mice exhibit dilated cardiomyopathy

The cardiac phenotype in 6 month old *mdx* mice is mild, but may be aggravated by exercise^33^,^34^. A forced exercise regimen was undertaken in control wild type C57BL/10 mice (*n* = 10) and *mdx* mice both untreated (*n* = 9) and treated with Pip6f-PMO (*n* = 10). From 12 weeks of age, mice were exercised on a treadmill 3 times every 2 weeks for 45 minutes (Supplementary Fig. 2). 10 *mdx* and 10 C57BL/10 unexercised mice were used as controls to monitor the effects of exercise alone. *Mdx* mice failed to run consistently, as they stopped intermittently and required constant encouragement (Supplementary Video 1). Indeed, 3 untreated *mdx* mice were removed from the study as they exhibited severe aversions to running, thereby reducing the sample size from 12 to 9 mice, which demonstrates their poor running capacity. C57BL/10 mice were markedly more active than their *mdx* counterparts (Supplementary Video 2).

**Figure 2:**
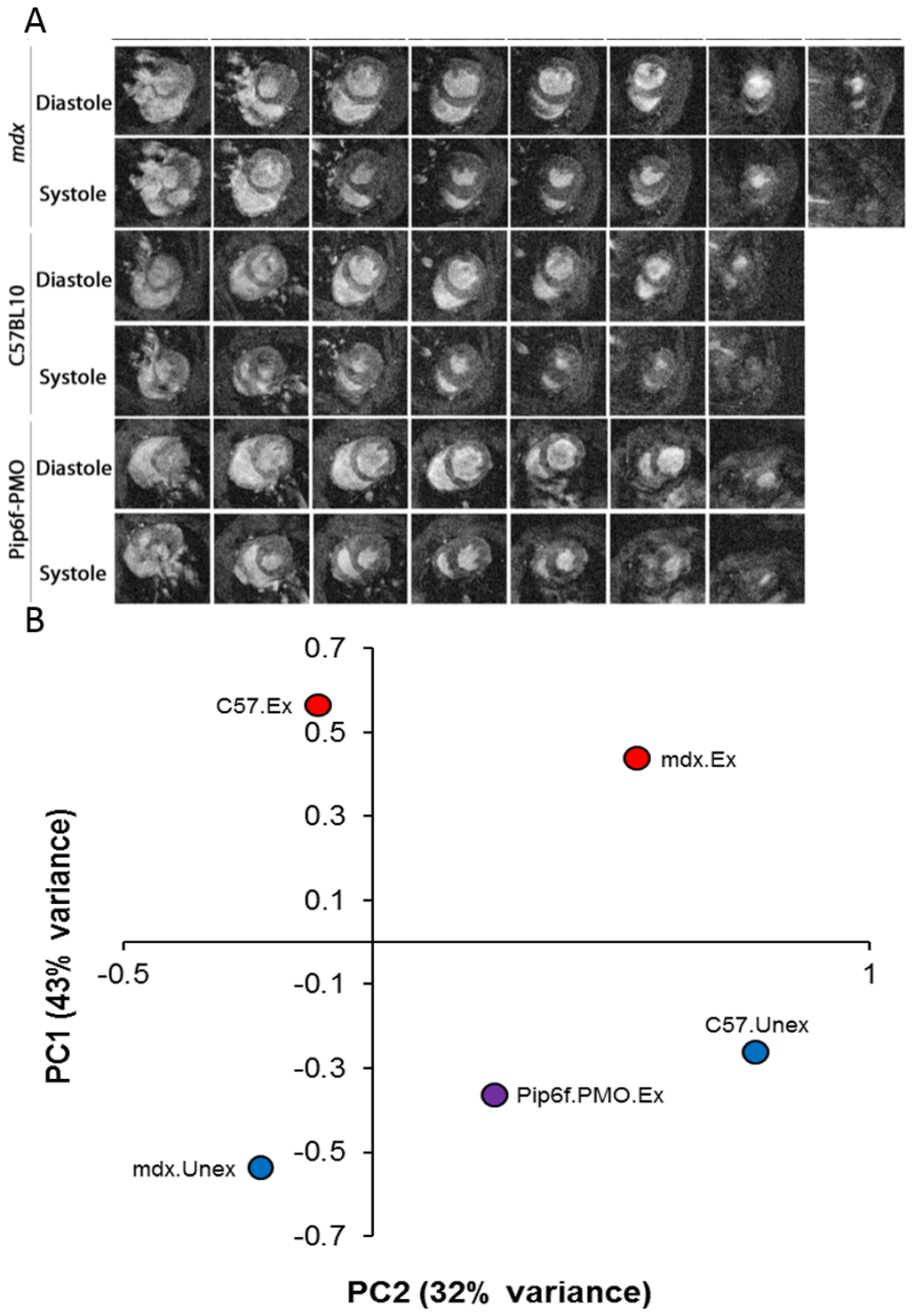
Contiguous cine-MRI images and correlation plot of cardiac function parameters in exercised *mdx* mice following Pip6f-PMO treatment. (*A*) Series of contiguous images at 1 mm increments throughout the entire hearts of C57BL/10, *mdx* and Pip6f-PMO treated mice during diastole and systole (exercised cohorts only). Images displays significantly larger hearts for *mdx* untreated cohort. (*B*) Correlation plot of cardiac function parameters measured by cine-MRI in exercised *mdx* mice following Pip6f-PMO treatment. Principal component analysis plot of all left and right ventricle cardiac function parameters. The two components demonstrate the highest percentage of variance on the Y-axis (component 1, 43%) and X-axis (component 2, 32%). This plot shows how treated mice tend to display values of the component 1 intermediate between C57BL/10 and *mdx* mice and values of the component 2 corresponding to unexercised mice.

All groups of mice underwent cine-MRI at 24 weeks of age, 5 days after the last exercise event in the exercised groups. A range of cardiac parameters were determined and all normalised to body weight with the exception of ejection fraction (EF) (Table 1). Data was analysed using stringent statistical tests (two-way ANOVA and one-way ANOVA with Tukey post-hoc and Games-Howell post-hoc). As previously described, most LV parameters were unchanged between the unexercised *mdx* and C57BL/10 cohorts at 24 weeks of age (with the exception of LV cardiac output; LV cardiac output (CO))^28^,^35^. The exercise regimen was developed to moderately increase the workload on the heart without causing damage to cardiac tissue. The exercise had a significant effect on the untreated *mdx* hearts for a number of cardiac measurements (increased average mass, end diastolic volume and end systolic volume with consequential decrease in LV EF; interaction effect between exercise and mouse groups determined by two-way ANOVA - see Table 1). These changes suggest reduced contractility of the heart and the onset of cardiac hypertrophy. This is complemented by Fig. 2 A which illustrates thicker LV walls of the *mdx* exercised cohort in scans 4–6 at systole. The heart rate was also elevated, with resultant increase in CO, suggesting compensatory mechanisms to increase cardiac contractility (Table 1).

These results are complemented by the one-way ANOVA analysis whereby the *mdx* and C57BL/10 exercised cohorts were directly compared (*mdx* mice: LV ESV larger, with reduced LV EF, RV EDV and ESV larger with consequential decrease in EF; Table 1).

### Pip6f-PMO administration prevents onset of cardiomyopathy

While exercise induced a strong heart phenotype in the *mdx* mice, treatment with Pip6f-PMO stabilised cardiac function, as demonstrated by cine-MRI. Moreover, in contrast to the untreated exercised *mdx* cohort which required much encouragement, the Pip6f-PMO group ran very well (Supplementary Video 3). The most notable cine-MRI result was the significantly larger average mass of the heart for the exercised *mdx* cohort compared to the Pip6f-PMO treated group (*P* < 0.05; Fig. 2A and Table 1). All LV and most of the RV cardiac parameters of the Pip6f-PMO treated cohort were comparable to the C57BL/10 cohort (Table 1). In addition, the Pip6f-PMO treated cohort and the unexercised *mdx* cohort (Table 1) exhibited very similar cardiac parameter values. Indeed, when all LV and RV MRI parameters were correlated using a principal component plot, the treated cohort exhibits a similar profile to the C57BL/10 and *mdx* unexercised cohorts (Fig. 2B). Overall the untreated, exercised *mdx* mouse cohort developed a dilated and compensated cardiomyopathy; however Pip6f-PMO treatment prevented hypertrophy and prevented exercise induced deterioration of cardiac function in *mdx* hearts.

### Reduction of cardiac damage markers and heart pathology in Pip6f-PMO treated mice

It was anticipated that the exercise-induced increase in cardiac workload combined with the absence of dystrophin in the *mdx* heart would result in an increase in tissue biomarkers of cardiac injury or damage.

**Figure 3:**
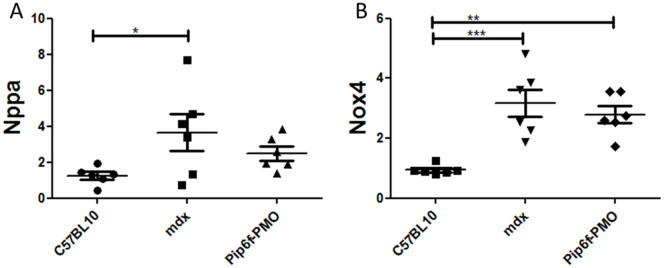
Gene expression analysis for markers of cardiac injury in exercised *mdx* mice following Pip6f-PMO treatment. RT-qPCR analysis of *Nppa* (*A*) and *Nox4* (*B*) in heart tissue normalised to C57BL/10 cohort. The untreated *mdx* exercised cohort reveals elevated expression of these injury markers whereas there is partial normalisation for the Pip6f-PMO cohort. Statistical significance was determined using ANOVA followed by Tukey post-hoc test (*** = *P* < 0.001, ** = *P* < 0.01, * = *P* < 0.05).

Two such markers, *Nppa* and *Nox4*, were measured 5 days following exercise in *mdx* mouse hearts. Atrial natriuretic peptide (Nppa) is secreted by the atria in response to haemodynamic overload or stress^36^. Its release results in the reduction of blood pressure and cardiac hypertrophy and is therefore considered a compensatory mechanism^36^ but also serves as a diagnostic measure of heart failure^37^. Quantitative assessment (RT-qPCR) revealed elevated expression of this gene in the untreated exercised *mdx* group which was significantly higher than in the C57BL/10 cohort (*P* < 0.05; Fig. 3A). The expression of *Nppa* in the Pip6f-PMO treated cohort was partially normalised.

NADPH oxidase 4 (Nox4) has been associated with increases in oxidative stress^38^ and has previously been described in *mdx* mouse studies to be associated with cardiac dysfunction^39^. *Nox4* expression in the *mdx* exercised cohort was markedly elevated compared to C57BL/10 mice (*P* < 0.001; Fig. 3B). *Nox4* levels in the Pip6f-treated cohort remained elevated, but to a lesser extent than in the *mdx* group (*P* < 0.01).

**Figure 4:**
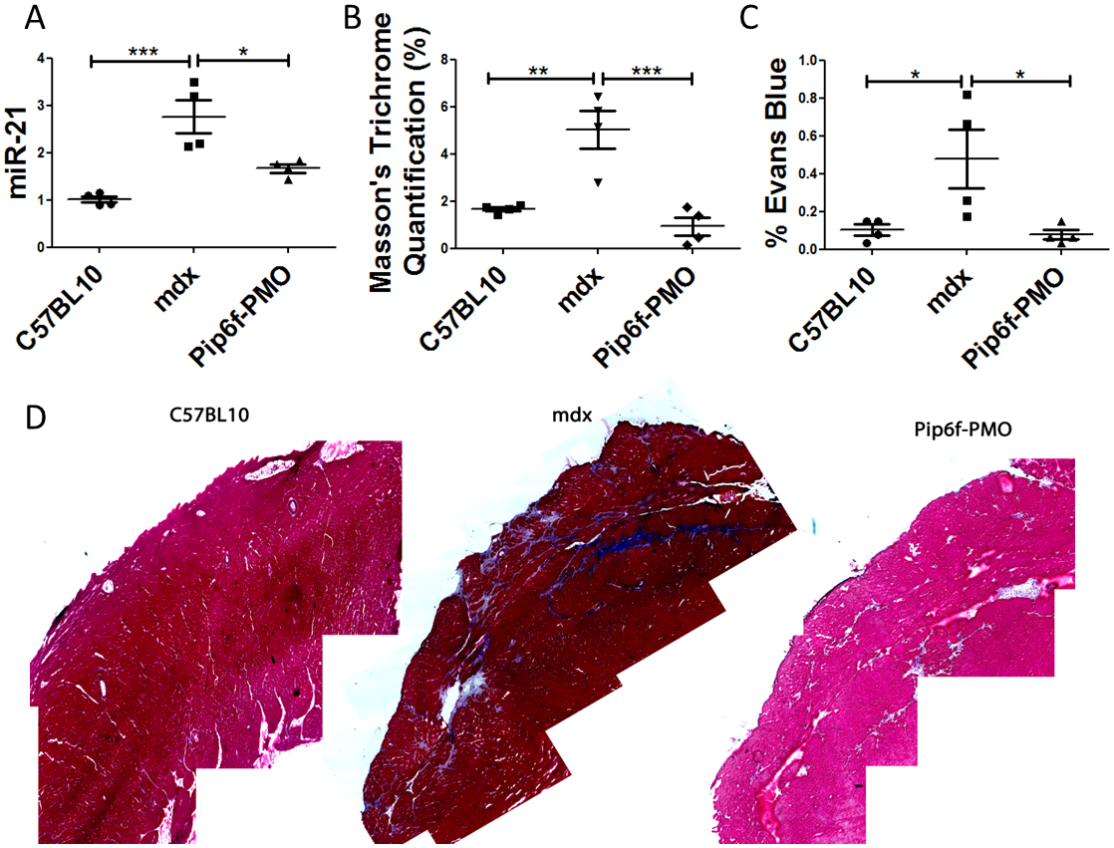
Reduction in cardiac pathology as determined by the detection of fibrosis and sarcolemmal damage in the hearts of exercised *mdx* mice following Pip6f-PMO treatment. (*A*) RT-qPCR analysis of miRNA-21 in heart tissue normalised to C57BL/10 cohort. (*B*) Quantification of Masson's trichrome staining in hearts of exercised cohorts. (*C*) Evans blue dye infiltration into heart following exercise. (*D*) Masson's trichrome images of worst areas of collagen deposition. miRNA-21 expression and Evans blue dye leakage of Pip6f-PMO hearts is normalised in contrast to untreated control. In addition Masson's trichrome staining is reduced and the representative images indicate less fibrosis then the untreated *mdx* cohort. Statistical significance was determined using ANOVA followed by Tukey post-hoc test (*** = *P* < 0.001, ** = *P* < 0.01, * = *P* < 0.05).

MicroRNA (miRNA) 21 is another biomarker of cardiac injury, specifically, it has been directly linked to myocardial fibrosis during hypertrophy^40^. *Mdx* mice showed an increase in miR-21 expression over wild type mice suggesting an increase in interstitial fibrosis (*P* < 0.001; Fig. 4A). Treatment with Pip6f-PMO partially normalised miR-21 expression (*P* < 0.05) over the untreated counterpart. The quantification of Masson's trichrome staining, which stains for collagen deposition, confirmed this observation of significantly reduced interstitial fibrosis in Pip6f-PMO treated *mdx* hearts (Fig. 4B). Again the untreated *mdx* hearts exhibited greater collagen deposition compared to C57BL/10 (*P* < 0.01) and Pip6f-PMO (*P* < 0.001) cohorts. Images of cardiac fibrosis strongly indicated the greater collagen deposition in untreated *mdx* hearts that was significantly reduced by treatment (Fig. 4D). Moreover, infiltration of Evans blue dye in the hearts of Pip6f-treated mice was also significantly reduced compared to untreated counterparts (*P* < 0.05; Fig. 4C). This showed that Pip6f-PMO treatment prevented sarcolemmal damage following exercise. These results denote a significant reduction in cardiac pathology in the treated mouse cohort.

Blood analyses of markers for toxicity were performed to determine the safety of the Pip6f-PMO treatment regimen. Plasma toxicity markers, including markers of hepatic and renal function, were unaffected by Pip6f-PMO treatment (Supplementary Fig. 7).

## Discussion

While AO exon skipping clinical trials appear promising for DMD^17^,^18^,^20^,^41^,^42^, the high incidence of cardiomyopathy in these patients, with associated high levels of mortality, indicate that restoring dystrophin protein in heart to modulate the cardiac phenotype is of high priority. Here we evaluated the therapeutic value of a highly potent peptide-PMO, Pip6f-PMO, which we previously demonstrated to be active in heart^30^. Using a forced exercise model we show that repeat treatment with Pip6f-PMO induced high levels of dystrophin restoration in heart which prevented cardiac hypertrophy and stabilised other cardiac parameters as determined by cine-MRI. Moreover, we also show that treatment ameliorates cardiac pathology as evidenced by reduced collagen and interstitial fibrosis and improved sarcolemmal integrity. These results suggest that high levels of dystrophin protein restoration in heart early in the course of DMD disease progression might have a significant clinical benefit.

A major limitation of the *mdx* mouse model of DMD is the mild cardiomyopathy which typically manifests only with advanced age^35^. Whilst previous peptide-PMO studies have shown dystrophin restoration in heart and some influence on cardiac function parameters, they either required dobutamine administration to stress the heart of young *mdx* mice^27^ or observed improvements predominantly in the RV of 6 month old mice^28^. Dobutamine administration is commonly used to exacerbate cardiac dysfunction in *mdx* mice, however this acute insult does not model the effect of progressive macro-structural changes to the myocardium typical of a degenerative cardiomyopathy. In order to induce a cardiomyopathy which recapitulates the DMD patient phenotype, mice were subjected to a moderate exercise protocol over a 12 week period. This exercise regimen was based on the ‘Treat NMD protocol and guidelines' for exercising *mdx* mice (http://www.treat-nmd.eu). As this protocol has only previously been utilised in assessing skeletal muscle pathology, the exercise regimen was modified. The established exercise regimen was designed so as to cause minimal damage to the heart while moderately increasing the cardiac workload in order to advance the stage of cardiomyopathy incrementally. This 12 week time-course was sufficient to yield significant functional changes in the untreated *mdx* heart (Fig. 2 and Table 1). Treadmill running was favoured over voluntary running, since wild-type mice are known to run further distances than *mdx* mice^33^,^43^,^44^.

Cine-MRI is considered the optimal technique for cardiac function assessment in small animals^45^. Cine-MRI demonstrated that moderate forced exercise was capable of inducing dilated cardiomyopathy in *mdx* mice, characterised by an enlarged heart and reduced contractility. This condition, dilated cardiomyopathy, is exhibited in all DMD patients by the age of 18 years^3^,^46^. However, treatment with Pip6f-PMO prevented these events. The low dose administration regimen of Pip6f-PMO induced high dystrophin protein restoration levels in skeletal muscle and approximately 28% dystrophin protein restoration in the heart. This level of dystrophin prevented hypertrophy, with left and right ventricular volumes in the normal range. In addition, LV CO and RV CO were stable. This study indicates that approximately 28% dystrophin restoration is sufficient to prevent the onset of exercised-induced dilated cardiomyopathy and maintain cardiac function.

In addition to MRI changes, the untreated exercised *mdx* cohort exhibited elevated markers of oxidative damage and injury, and fibrosis (collagen deposition) and sarcolemmal damage in the heart. This compares well with previous studies that show clear myocardial changes following exercise in *mdx* mice^33^,^34^,^47^. In contrast, the Pip6f-PMO treatment prevented injury in the *mdx* heart. It further reduced cardiac pathology as shown by significant reductions in cardiac fibrosis and sarcolemmal damage which demonstrated the protective capacity of a highly effective dystrophin restoration treatment. Cardiac fibrosis generally correlates with the decline in cardiac function^48^,^49^ and therefore the marked reduction of myocardial fibrosis would likely have had a beneficial effect on cardiac function.

Our data suggests that restoring high levels of dystrophin protein in heart is likely to prevent the progression of cardiomyopathy in DMD patients. This supports the idea that DMD patients should be treated as early as possible. It has been reported that mild molecular abnormalities in *mdx* heart manifest as early as 10–12 weeks of age and therefore it is likely that our treatment was not initiated prior to the onset of disease^50^,^51^. It is possible that an even greater degree of cardio-protection would have been observed if Pip6f-PMO treatment had been initiated earlier, in younger *mdx* mice. In addition, this work demonstrates ‘proof of principle' for the utilisation of Pip6-conjugated AOs for the correction of cardiac structural protein defects. Three genes encoding cardiac sarcomeric proteins, MYH7, MYBPC3 and TNNT2 have been implicated in over 70% of all familial hypertrophic cardiomyopathies^52^ and indeed exon skipping technology has already been demonstrated as a possible therapy for *Mbpc3* mutations^53^. Thus Pip-PMO compounds could have wider application in the treatment of many familial cardiac defects.

## Methods

### Synthesis of Peptide-PMO Conjugates

Pip6f Ac-(RXRRBRRXRFQILYRXRBRXRB)-COOH, where X is aminohexanoic acid and B is β-alanine) was synthesized by standard solid phase Fmoc chemistry and purified by HPLC. The PMO sequence (5'-GGCCAAACCTCGGCTTACCTGAAAT-3') was purchased from Gene Tools LLC. Pip6f was conjugated to PMO through an amide linkage at the 3' end of the PMO, followed by purification by HPLC as previously described^30^. The final product was analysed by MALDI-TOF MS and HPLC. Peptide-PMO conjugates were dissolved in sterile water and filtered through a 0.22 μm cellulose acetate membrane before use.

### Animals and Intravenous Injections

Male C57BL/10 mice were attained from Harlan Laboratories (Oxford, UK) and *mdx* mice were bred in house at the Biomedical Sciences Unit (University of Oxford, UK). All procedures were authorized and approved by the University of Oxford ethics committee and UK Home Office (project licence 30/2907). Procedures were carried out in the Biomedical Sciences Unit, University of Oxford, in accordance with ‘Laboratory Animal Handbooks NO.14; The Design of Animal Experiments (2010)'. For the treated cohort, 12 week old male *mdx* mice were injected via the tail vein with Pip6f-PMO prepared in 0.9% saline solution at a dose of 10 mg/kg. Mice were injected daily for 4 days followed by additional administrations every two weeks; 9 doses in total.

### Exercise Regimen

This exercise regimen was modified from a Treat-NMD protocol (http://www.treat-nmd.eu/downloads/file/sops/dmd/MDX/DMD_M.2.1.001.pdf). The Exer 3/6 treadmill (Columbus Instruments, USA) was used for the exercised cohorts. Exercise commenced at 12 weeks of age and mice were exercised 3 times every 2 weeks for a 12 week period. Mice were allowed 2 minutes for familiarisation. The exercise regimen was started at 5 m/min and gradually increased in 1 m/min increments to 10 m/min for the first 2 exercise days. For the following 6 exercise sessions, mice were run for 45 minutes with the speed of the treadmill incrementally increased to 12 m/min. The mice were run at 12 m/min for 45 minutes for the remaining exercise sessions.

### Cine-MRI

All mice underwent cardiac cine-MRI at 24 weeks of age, as previously described^35^. Mice were anaesthetised and placed in the supine position into a purpose built cradle. ECG electrodes were inserted into the forepaws of the mouse and the respiration loop was taped across the abdomen. Once the mouse was secured, with a stable ECG measurement, the cradle was lowered into a vertical-bore, 11.7 T MR system (Magnex Scientific, Oxon, UK) with a 40 mm birdcage coil (Rapid Biomedical, Wurzburg, Germany). Images were acquired using a Bruker console running Paravision 2.1.1 (Bruker Medical, Ettlingen, Germany). The entire left and right ventricles were imaged by taking a contiguous stack of cine images in 1 mm increments. Images were analysed using ImageJ software (NIH Image, Bethesda, MD). The epicardial and endocardial borders were outlined using the ImageJ free-hand tool at end-diastole and end-systole.

Following MRI, mice were sacrificed by CO_2_ inhalation, and muscles and other tissues harvested and snap-frozen in cooled isopentane before storage at −80°C.

### Immunohistochemistry and Quantification

Transverse sections of tissue samples were sectioned (8 μm thick) and co-stained with rabbit-anti-dystrophin (Abcam) and rat anti-laminin (Sigma) as previously described^30^. Dystrophin was quantified relative to laminin co-stain using ImagePro software (MediaCybernetics) and normalised to C57CL/10. Briefly, 4 images of dystrophin and the corresponding laminin fields were taken for each section. Following the Arechavala-Gomeza approach^54^, 10 regions of interest were randomly placed on the laminin image which was overlaid on the corresponding dystrophin image to attain the minimum and maximum fluorescence intensity (Image-Pro, Media Cybernetics, Inc.). Data was normalised relative to C57BL/10. The percentage recovery score was calculated using the following equation: (dystrophin recovery of treated *mdx* mice-dystrophin recovery of untreated *mdx* mice)/(dystrophin recovery of C57BL/10 mice-dystrophin recovery of untreated *mdx* mice); as described on the TREAT-NMD website (http://www.treat-nmd.eu/downloads/file/sops/dmd/MDX/DMD_M.1.1_001.pdf).

### RT-PCR and RT-qPCR for Mouse Tissues

Total RNA was extracted using TRIzol reagent (Invitrogen) following manufacturer's instructions. For the RT-PCR reaction, 400 ng of RNA template was used in a 50 μl reverse transcription reaction using One Step RT-PCR Kit (QIAGEN) and gene specific primers as previously described^30^. Two microlitres of cDNA was amplified in a 50 μl nested PCR (QIAGEN PCR kit).

For RT-qPCR, 0.5–1 μg of RNA was reverse transcribed using a High Capacity cDNA Synthesis kit (Applied Biosystems). Gene specific primers sets (IDT) for markers of damage (*Nppa-* Assay Mm.PT.58.8820983 and *Nox4*- Assay MM.PT.58.12973594.g) were amplified using TaqMan Universal mastermix (Applied Biosystems) run on the StepOne Plus Real-Time PCR system (Applied Biosystems). The quantity mean values for each gene were normalised relative to the reference gene, *Ywhaz* (Assay Mm.PT.39a.22214831). For *Dmd* transcript levels, 25 ng of cDNA template was amplified using Taqman Gene Expression Master Mix. Levels of *Dmd* exon 23 skipping were determined by multiplex qPCR of FAM-labelled primers spanning Exon 20–21 (Assay Mm.PT.47.9564450, Integrated DNA Technologies) and HEX-labelled primers spanning Exon 23–24 (Mm.PT.47.7668824, Integrated DNA Technologies). The percentage of *Dmd* transcripts lacking exon 23 was determined by normalising *Dmd* exon 23–24 amplification levels to *Dmd* exon 20–21 levels.

miRNA qPCR was performed as previously described^55^. To quantify mature miRNA abundance, small RNA TaqMan Assays (Applied Biosystems) were used according to manufacturer's instructions. As described previously^56^, 5 ng of total RNA was reverse transcribed using the TaqMan miRNA Reverse Transcription Kit (Applied Biosystems). 1.33 μl of RT reaction were used in each qPCR and miR-16 was served as endogenous normalizer miRNA.

### Protein Extraction and Western Blot

Samples were homogenised and quantified by Bradford assay (Sigma) as previously described^30^. Ten micrograms of untreated and treated *mdx* protein, and 5 μg and 2.5 μg of C57BL/10 protein (positive control) were loaded onto 3–8% Tris-Acetate gels (Invitrogen). Protein was blotted onto PVDF membrane and probed for dystrophin using DYS1 (Novocastra) and loading control, vinculin (Sigma). Primary antibodies were detected using IRDye 800 CW goat-anti mouse IgG (Licor). Western blots were imaged (LiCOR Biosciences) and analysed using the Odyssey imaging system. Western blots were quantified by calculating dystrophin protein expression relative to vinculin loading control.

### Masson's Trichrome Staining

Longitudinal sections of heart samples were cut (8 μm thick) and stained with a Masson's trichrome kit following the manufacturer's instructions (Sigma). Whole sections were imaged and reassembled. Fibrosis was quantified as a percentage of surface area stained with Masson's trichrome measured using ImageJ.

### Evans Blue Dye Administration

Between 3 and 4 mice from each cohort were injected intra-peritoneally with 1% Evans blue dye (10 μl/g of mouse; Sigma) and heart tissues were harvested 20 hours later. Hearts were sectioned (8 μm), placed in acetone for 10 minutes, washed with PBS and cover slipped. Evans blue infiltration was visualised using a Leitz DM RBE fluorescent microscope (Leica) and the entire section was imaged using Axiovision Rel 4.7 Software (Zeiss). The images were collated so that the entire section could be observed and the surface area of Evans blue staining was quantified using the threshold function of ImageJ software.

### Clinical Biochemistry

Plasma samples were extracted from the jugular vein of *mdx* mice immediately after sacrifice by CO_2_ inhalation. Analysis of toxicity biomarkers was performed by a clinical pathology lab, Mary Lyon Centre, MRC, Harwell, UK.

### Statistical Analysis

All values reported are mean ± standard error of the mean (SEM). Regarding cine-MRI data, each cohort was directly compared using Students t-test. microRNA RT-qPCR of murine heart tissues. Statistical significance was calculated with IBM SPSS Statistics (Version 22.0) using one- way ANOVA followed by Tukey post-hoc test (TU), or Games-Howell post-hoc test to correct for variance heterogeneity. Variance heterogeneity was assessed by Levene's tests. Two-way ANOVA was also performed to determine the interaction effect between exercise and the mouse groups (ie. *mdx* and C57BL/10). Principal component analysis was conducted on a correlation matrix using the function prcomp() in R (http://www.r-project.org/).

## Author Contributions

C.A.B., A.F.S., M.J.G. and M.J.A.W. designed the research. C.A.B., C.A.C., S.M.H., A.M.L.C.S., C.G. and G.M. performed the research. C.A.B., C.A.C., M.A.V., T.C.R. and K.C. analysed the data and C.A.B. and M.J.A.W. wrote the paper.

## Supplementary Material

Supplementary Information Supplementary Information

Supplementary Information Supplementary Video 1

Supplementary Information Supplementary Video 2

Supplementary Information Supplementary Video 3

## Figures and Tables

**Table 1:** Cardiac function parameters measured by cine-MRI in all cohorts. Table listing the mean values for all left and right ventricle cardiac parameters and standard error of the mean (SEM). Parameters were normalised to body weight. Note: The exceptions to this is the ejection fraction which is represented as a percentage and heart rate calculated as beats per minute (BPM). Weights are in grams. Statistical significance values were determined using one-way ANOVA followed by Tukey post-hoc test (TU), or Games-Howell (GH) post-hoc test to correct for variance heterogeneity. Each cohort is compared to all other cohorts

		One-way ANOVA statistical significance										Two-way ANOVA
		C57 Unex	SEM	C57 Ex	SEM	*mdx* Unex	SEM	*mdx* Ex	SEM	Pip6f-PMO Ex	SEM	Interaction effect between exercise and mouse group
Left ventricle/body weight	Average Mass^GH^	3.75	±0.20	3.45^/^	±0.07	3.43^/^	±0.08	3.91^~#+^	±0.11	3.45^/^	±0.05	0.002
	End Diastolic Volume^TU^	1.96	±0.14	1.67	±0.06	1.77	±0.05	2.10	±0.20	1.88	±0.03	0.007
	End Systolic Volume^TU^	0.74	±0.12	0.50^/^	±0.04	0.65	±0.08	0.90^+^	±0.15	0.71	±0.04	0.02
	Stroke Volume^GH^	1.22	±0.04	1.17	±0.05	1.12	±0.04	1.20	±0.06	1.17	±0.03	N/S
	Cardiac output^TU^	0.52^#^	±0.02	0.48	±0.02	0.42^/^*	±0.03	0.52^#^	±0.02	0.44	±0.01	0.003
Left ventricle%	Ejection Fraction^TU^	63.82	±2.65	70.08^/^	±2.26	63.98	±3.59	58.56^+^	±2.48	62.47	±1.93	0.04
Right ventricle/body weight	End Diastolic Volume^TU^	1.64	±0.04	1.52^/^	±0.08	1.53^/^	±0.05	1.79^+#^	±0.08	1.68	±0.06	0.004
	End Systolic Volume^TU^	0.45^//^	±0.02	0.38 ^##///~^	±0.03	0.55^++^	±0.03	0.66**^+++^	±0.05	0.54^+^	±0.04	0.009
	Stroke Volume^GH^	1.18^#^	±0.04	1.14	±0.07	0.98*	±0.04	1.13	±0.05	1.14	±0.03	0.05
	Cardiac output^TU^	0.50^###^	±0.02	0.47^#^	±0.03	0.37***^//+^	±0.03	0.49^##^	±0.02	0.43	±0.01	0.001
Right ventricle %	Ejection Fraction^TU^	72.23^##//^	±1.01	74.87^###///~^	±1.65	64.36**^+++^	±1.74	63.34**^+++^	±1.65	68.35^+^	±1.24	N/S
BPM	Heart Rate^TU^	427.02	±15.42	410.02	±11.04	373.64	±20.22	433.28	±12.42	376.72	±13.21	0.03
Gram	Body weight^GH^	32.40	±0.77	31.00^/~~^	±0.33	32.40	±1.20	33.33^+^	±0.62	33.90^++^	±0.53	N/S

*significantly different to C57 Unex, + significantly different to C57 Ex, # significantly different to *mdx* Unex, / significantly different to *mdx* Ex and ~ significantly different to Pip6f-PMO Ex. Number of symbols denotes significance i.e. *** = *P* < 0.001, ** = *P* < 0.01, * = *P* < 0.05. Two-way ANOVA was also performed to determine interaction effects between exercise and mouse groups.
