# Supplementary material for: Prevention of exercised induced cardiomyopathy following Pip-PMO treatment in dystrophic mdx mice
Source: Sci Rep. 2015 Mar 11;5:8986. doi: 10.1038/srep08986 (PMC4355666; doi:10.1038/srep08986)
Supplement: Supplementary Information [file srep08986-s1.pdf]

Supplementary Information

Title:

Prevention of exercised induced cardiomyopathy following Pip-PMO treatment in dystrophic *mdx* mice

Authors:

Corinne A. Betts, Amer F. Saleh, Carolyn A. Carr, Suzan M. Hammond, Anna M. L. Coenen-Stass, Caroline Godfrey, Graham McClorey, Miguel A. Varela, Thomas C. Roberts, Kieran Clarke, Michael J. Gait, Matthew J. A. Wood

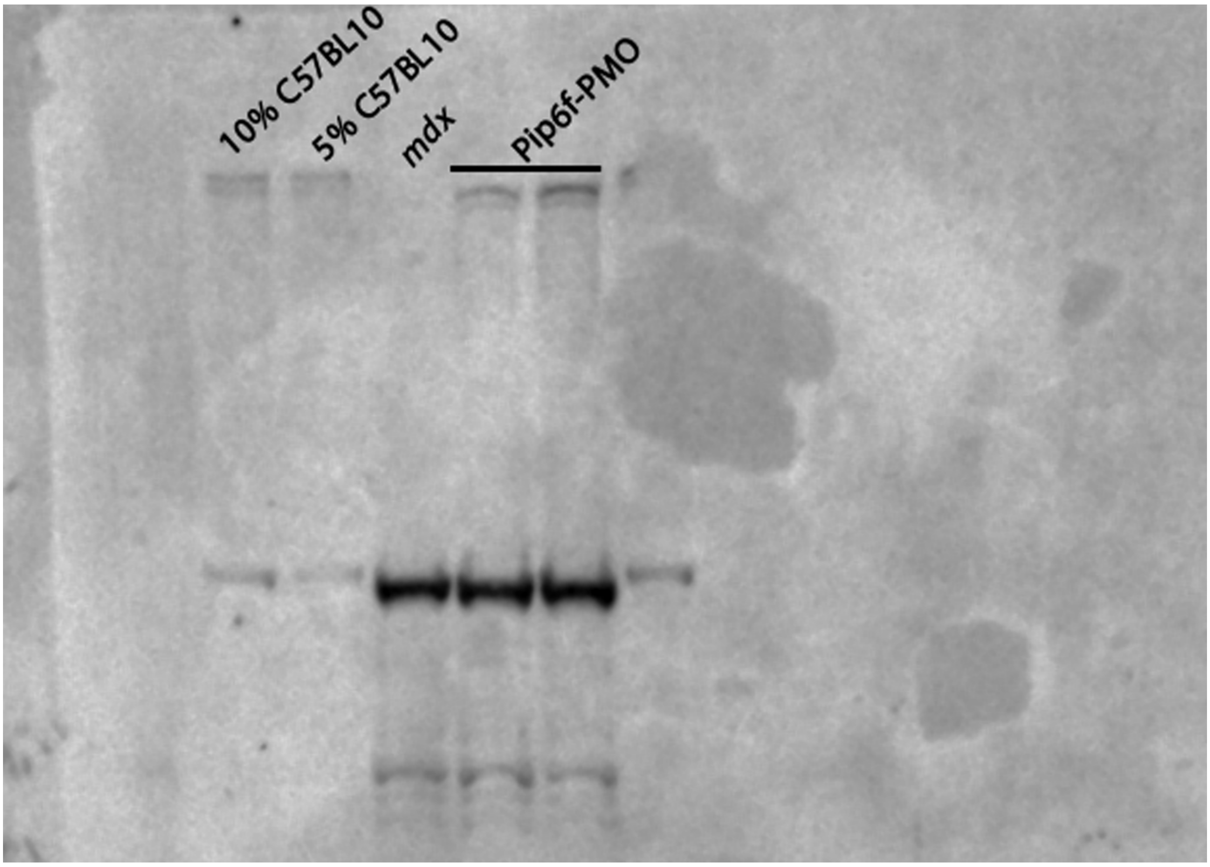
10% C57BL10
5% C57BL10
mdx
Pip6f-PMO

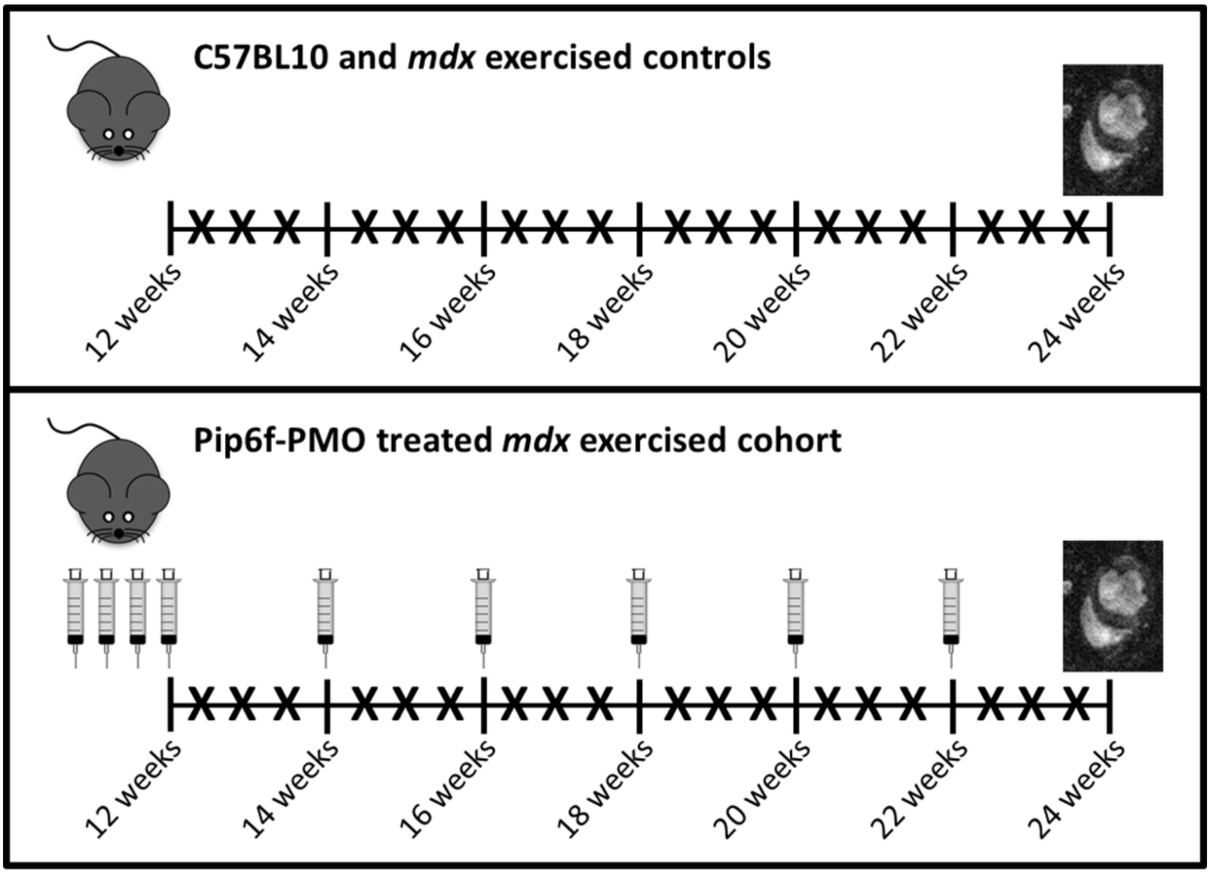

C57BL10 and *mdx* exercised controls
12 weeks
14 weeks
16 weeks
18 weeks
20 weeks
22 weeks
24 weeks
Pip6f-PMO treated *mdx* exercised cohort
12 weeks
14 weeks
16 weeks
18 weeks
20 weeks
22 weeks
24 weeks

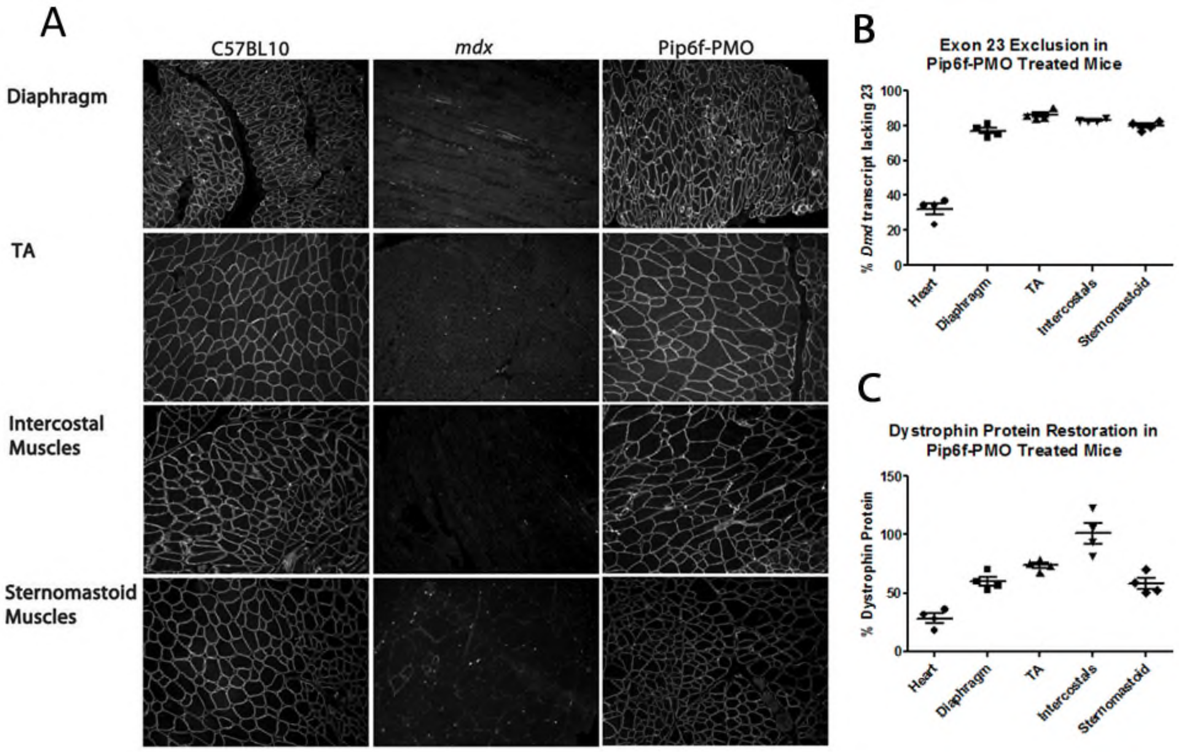

A
C57BL10
mdx
Pip6f-PMO
Diaphragm
TA
Intercostal Muscles
Sternomastoid Muscles
B
Exon 23 Exclusion in Pip6f-PMO Treated Mice
% Dmd transcript lacking 23
0
20
40
60
80
100
Heart
Diaphragm
TA
Intercostals
Sternomastoid
C
Dystrophin Protein Restoration in Pip6f-PMO Treated Mice
% Dystrophin Protein
0
50
100
150
Heart
Diaphragm
TA
Intercostals
Sternomastoid

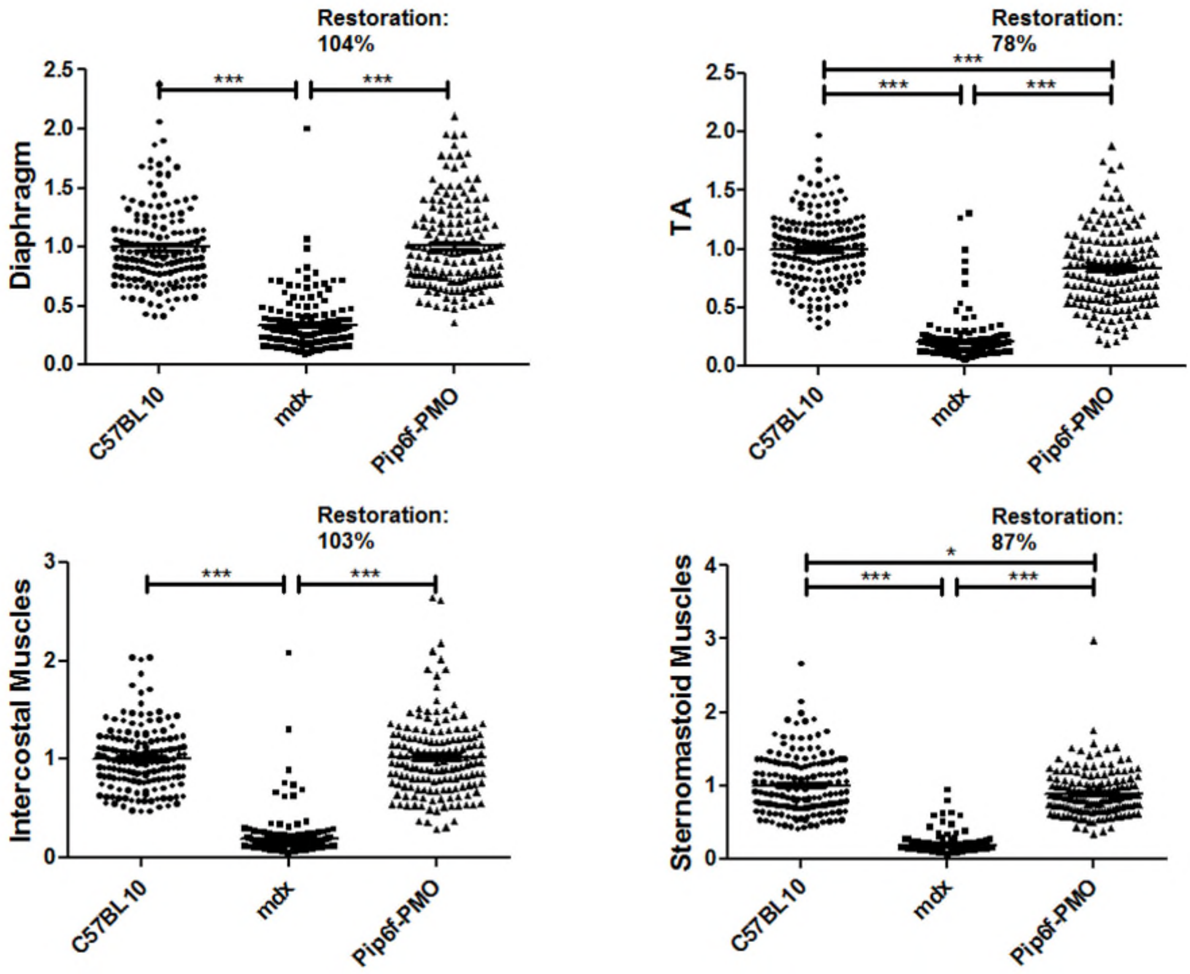
Restoration:
104%
Diaphragm
0.0
0.5
1.0
1.5
2.0
2.5
***
***
C57BL10
mdx
Pip6f-PMO
Restoration:
78%
TA
0.0
0.5
1.0
1.5
2.0
2.5
***
***
***
C57BL10
mdx
Pip6f-PMO
Restoration:
103%
Intercostal Muscles
0
1
2
3
***
***
C57BL10
mdx
Pip6f-PMO
Restoration:
87%
Sternomastoid Muscles
0
1
2
3
4
*
***
***
C57BL10
mdx
Pip6f-PMO

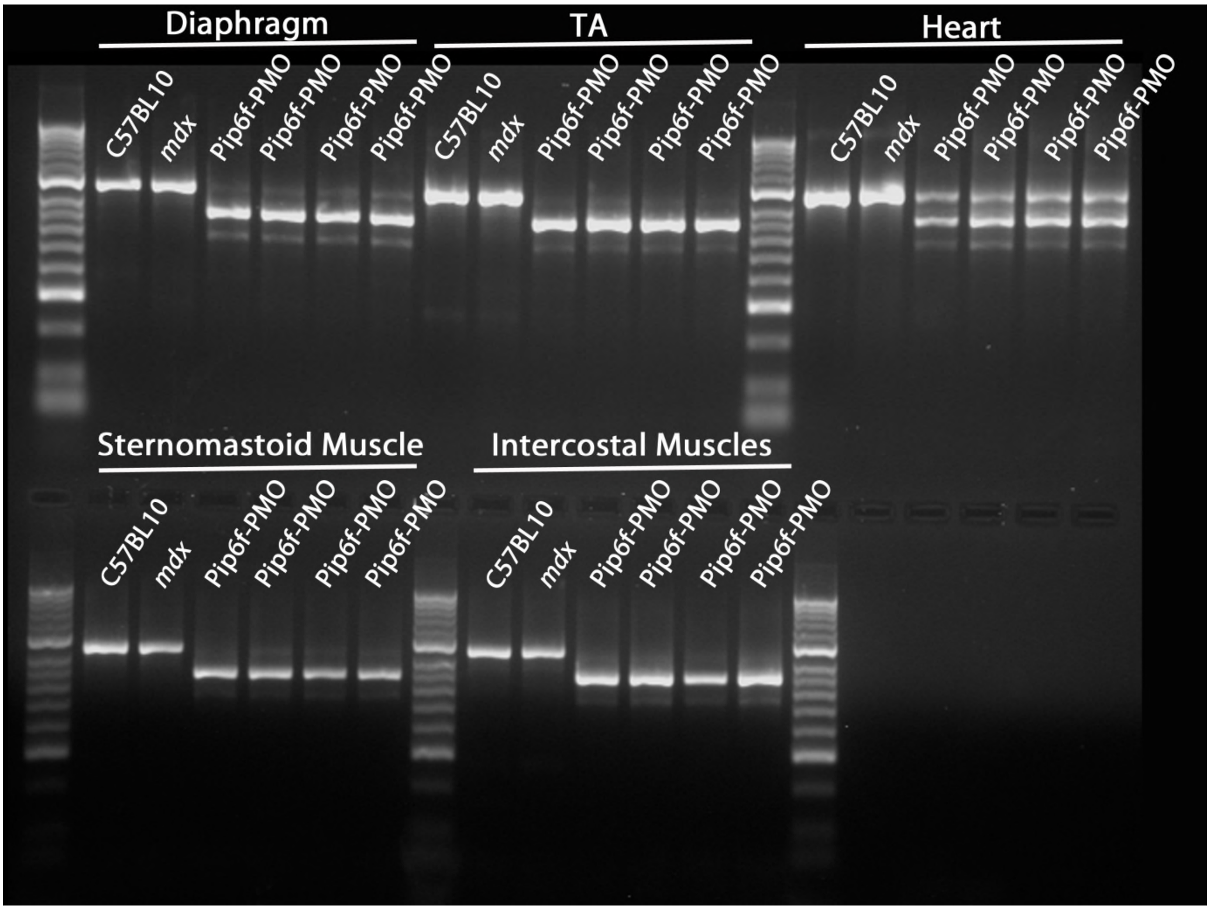

Diaphragm
TA
Heart
C57BL10
mdx
Pip6f-PMO
Pip6f-PMO
Pip6f-PMO
Pip6f-PMO
C57BL10
mdx
Pip6f-PMO
Pip6f-PMO
Pip6f-PMO
Pip6f-PMO
C57BL10
mdx
Pip6f-PMO
Pip6f-PMO
Pip6f-PMO
Pip6f-PMO
Sternomastoid Muscle
Intercostal Muscles
C57BL10
mdx
Pip6f-PMO
Pip6f-PMO
Pip6f-PMO
Pip6f-PMO
C57BL10
mdx
Pip6f-PMO
Pip6f-PMO
Pip6f-PMO
Pip6f-PMO

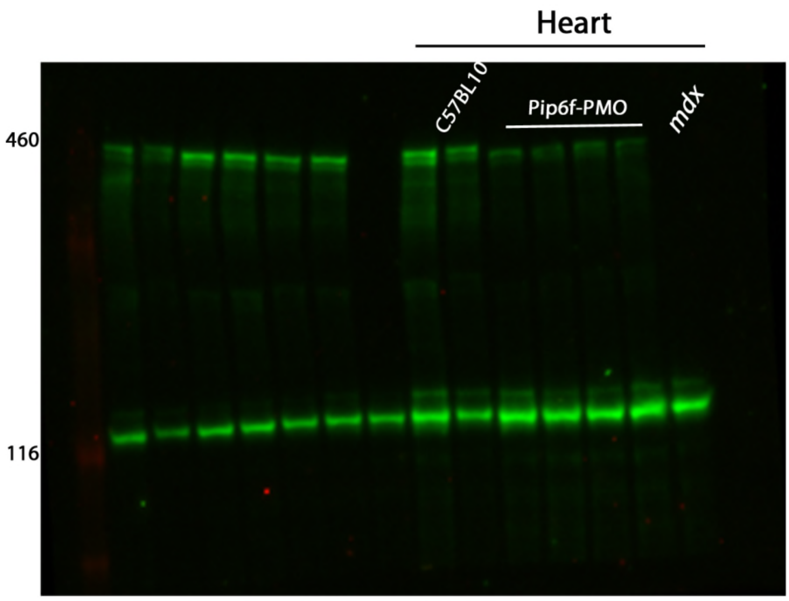
Heart
C57BL10
Pip6f-PMO
mdx
460
116

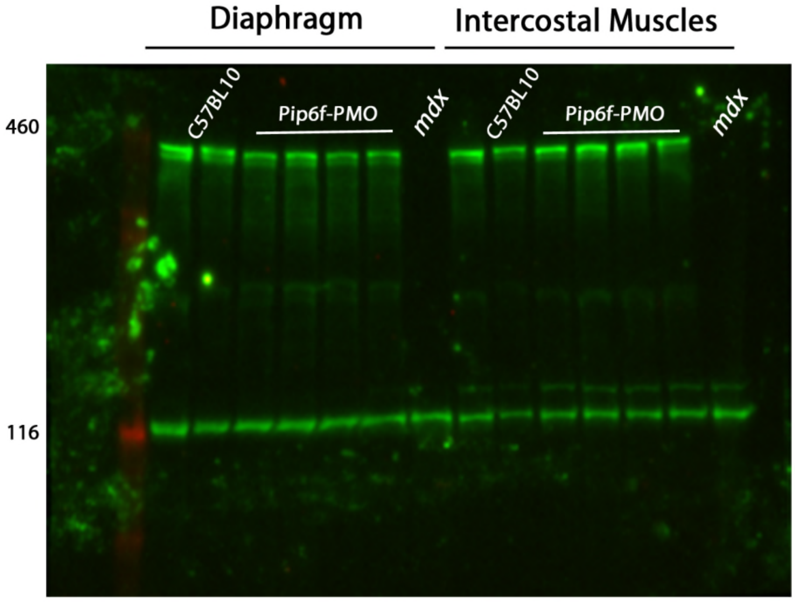
Diaphragm
Intercostal Muscles
C57BL10
Pip6f-PMO
mdx
C57BL10
Pip6f-PMO
mdx
460
116

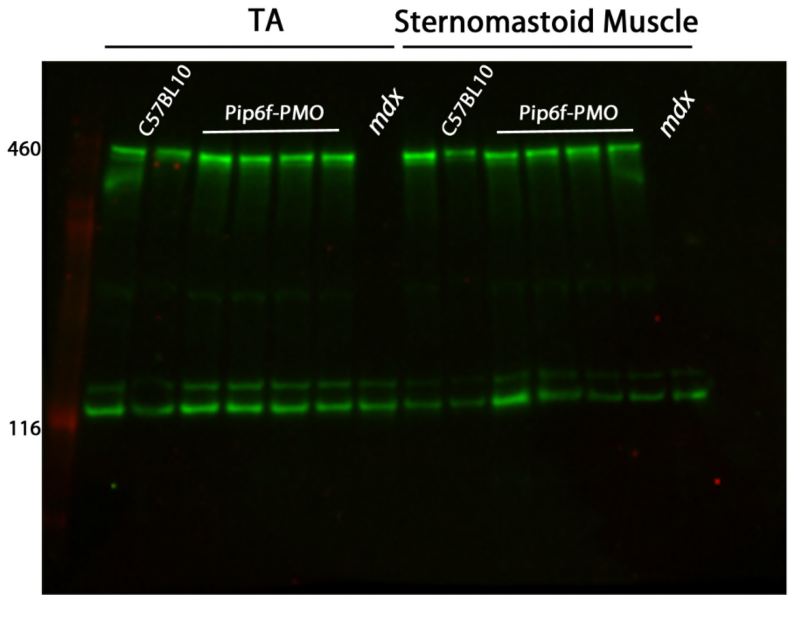
TA
Sternomastoid Muscle
C57BL10
Pip6f-PMO
mdx
C57BL10
Pip6f-PMO
mdx
460
116

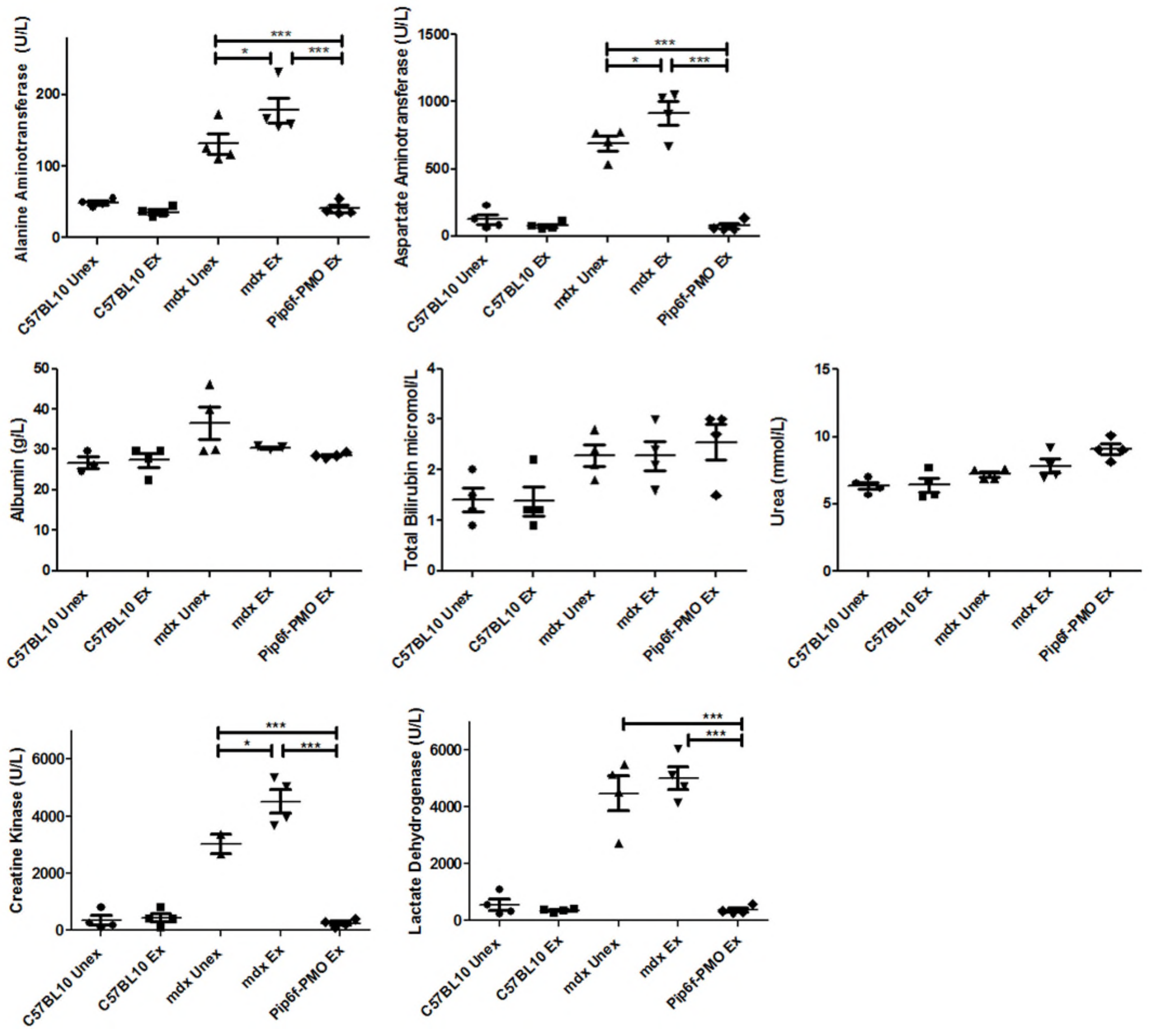

Alanine Aminotransferase (U/L)
Aspartate Aminotransferase (U/L)
Albumin (g/L)
Total Bilirubin micromol/L
Urea (mmol/L)
Creatine Kinase (U/L)
Lactate Dehydrogenase (U/L)
C57BL10 Unex
C57BL10 Ex
mdx Unex
mdx Ex
Pip6f-PMO Ex
*
***

Supplementary Figure Legends

Supplementary Fig. 1. Full-length image of western blot showing dystrophin protein expression and vinculin loading control in the hearts of C57BL/10, *mdx* untreated and Pip6f-PMO treated mice following a single administration. Cropped version in **Figure 1*B***.

Supplementary Fig. 2. Schematic illustrating the administration and exercise regimen for the study. The *mdx* and C57BL/10 exercised cohorts (top) commenced the exercise regime at 12 weeks of age and underwent a further 12 weeks of exercise with 3 bouts of 45 minute exercise every 2 weeks. The Pip6f-treated cohort received 4 daily administrations of 10 mg/kg Pip6f-PMO at 12 weeks of age, followed by administrations every 2 weeks. This cohort was exercised precisely the same as the untreated cohorts. Administration (injection symbol) and bouts of exercise (X) are indicated at the relevant time points. All mice underwent cine-MRI at 24 weeks of age.

Supplementary Fig. 3. Dystrophin expression and protein restoration in multiple skeletal tissues of exercised *mdx* mice following Pip6f-PMO treatment. (*A*) Representative image of dystrophin immunohistochemical staining in diaphragm, *tibialis anterior* (TA), intercostal muscles and sternomastoid muscles of exercised C57BL/10, *mdx* and Pip6f-treated *mdx* mice. (*B*) RT-qPCR graph illustrating the percentage of *Dmd* transcripts lacking exon 23, following normalisation to exon 20-21. This is shown for heart (32.3%, SEM 3.1), diaphragm (77.0%, SEM 1.8), TA (86.0%, SEM 1.3), intercostal muscles (83.0%, SEM 0.5) and sternomastoid muscles (79.8%, SEM 1.3) of Pip6f-PMO treated cohort. RT-PCR were also performed to illustrate Δ23 skipping for each tissue. For full RT-PCR agarose gels see **Supplementary Fig. 5**. (*C*) Graph illustrating quantification of western blots for Pip6f-PMO treated heart (28.5%, SEM 3.3) diaphragm (60.0%, SEM 3.35), TA (73.7%, SEM 1.9),

intercostal muscles (101.1%, SEM 7.6) and sternomastoid muscles (58.0%, SEM 4.0). For western blots, 10-15 µg of protein was loaded and dystrophin (dys) was quantified relative to vinculin loading control. Samples from each tissue were run under the same experimental conditions and on the same SDS gel. For full SDS western gels see **Supplementary Fig. 6**.

Supplementary Fig. 4. Immunohistochemical staining quantification in multiple skeletal tissues of exercised *mdx* mice following Pip6f-PMO treatment. Quantification of dystrophin protein in C57BL/10, *mdx* and Pip6f-PMO *mdx* mice for the diaphragm, *tibialis anterior* (TA), intercostal and sternomastoid muscles. Dystrophin expression is determined relative to laminin co-stain. Quantification is calculated using 120 regions of interest. This is calculated as the intensity value of dystrophin relative to the corresponding intensity value of laminin, normalised to C57BL/10 unexercised. The scatter plots show the normalised relative intensity values for each region of interest. Pip6f-PMO treatment resulted in widespread restoration of dystrophin expression in all tissues measured. Statistical significance was determined using ANOVA followed by Tukey post-hoc test (***=P <0.001, **= P <0.01*= P <0.05).

Supplementary Fig. 5. Full-length image of RT-PCR agarose gel showing Δ23 skipping in C57BL/10, *mdx* untreated and Pip6f-PMO treated mice following 12 week administration regimen. Heart, diaphragm, TA, intercostal muscles and sternomastoid muscles are shown.

Supplementary Fig. 6. Full-length images of western blots showing dystrophin protein expression and vinculin loading control in C57BL/10, *mdx* untreated and Pip6f-PMO treated

mice following 12 week administration regimen. Heart, diaphragm, TA, intercostal muscles and sternomastoid muscles are shown.

Supplementary Fig. 7. Liver and kidney toxicity markers and biomarkers of muscle integrity in plasma samples of exercised *mdx* mice following Pip6f-PMO treatment. Graphs showing the measurement of plasma alanine aminotransferase (ALT), aspartate aminotransferase (AST), albumin, total bilirubin, urea, creatine kinase (CK) and lactate dehydrogenase (LDH) in C57BL/10 control mice compared to *mdx* untreated and treated mice. Statistical significance was determined using ANOVA followed by Tukey post-hoc test (***=P <0.001, **= P <0.01*= P <0.05). Note: ALT and AST- C57BL/10 cohorts significantly different to both *mdx* cohorts; P <0.0001.

Supplementary videos

Supplementary Video 1. Representative video illustrating running behaviour of *mdx* mice undergoing forced running on a treadmill. Mice were run on an Exer3/6 treadmill for 45 minute bouts. *Mdx* mice did not run consistently and required constant encouragement.

Supplementary Video 2. Representative video illustrating running behaviour of C57BL/10 mice undergoing forced running on a treadmill. Mice were run on an Exer3/6 treadmill for 45 minute bouts. C57BL/10 mice ran well and consistently.

Supplementary Video 3. Representative video illustrating running behaviour of Pip6f-PMO treated mice undergoing forced running on a treadmill. Mice were run on an Exer3/6

treadmill for 45 minute bouts. Treated mice ran well and consistently and did not require encouragement unlike the untreated *mdx* cohort.
